# Geographical clustering of lung cancer in the province of Lecce, Italy: 1992–2001

**DOI:** 10.1186/1476-072X-8-40

**Published:** 2009-07-01

**Authors:** Massimo Bilancia, Alessandro Fedespina

**Affiliations:** 1 Department of Statistical Sciences "Carlo Cecchi", University of Bari, Via C. Rosalba n.53, 70124 Bari, Italy; 2 Department of Quantitative Methods and Economic Theory, University of Chieti "G. d'Annunzio", Viale Pindaro 42, 65127, Pescara, Italy

## Abstract

**Background:**

The triennial mortality rates for lung cancer in the two decades 1981–2001 in the province of Lecce, Italy, are significantly higher than those for the entire region of Apulia (to which the Province of Lecce belongs) and the national reference rates. Moreover, analyzing the rates in the three-year periods 1993–95, 1996–98 and 1999–01, there is a dramatic increase in mortality for both males and females, which still remains essentially unexplained: to understand the extent of this phenomenon, it is worth noting that the standardized mortality rate for males in 1999–01 is equal to 13.92 per 10000 person-years, compared to a value of 6.96 for Italy in the 2000–2002 period.

These data have generated a considerable concern in the press and public opinion, which with little scientific reasoning have sometimes identified suspected culprits of the risk excess (for example, the emission caused by a number of large industrial sites located in the provinces of Brindisi and Taranto, bordering the Province of Lecce). The objective of this paper is to study on a scientifically sound basis the spatial distribution of risk for lung cancer mortality in the province of Lecce. Our goal is to demonstrate that most of the previous explanations are not supported by data: to this end, we will follow a hybrid approach that combines both frequentist and Bayesian disease mapping methods. Furthermore, we define a new sequential algorithm based on a modified version of the Besag-York-Mollié (BYM) model, suitably modified to detect geographical clusters of disease.

**Results:**

*Standardized mortality ratios (SMRs) for lung cancer in the province of Lecce*: For males, the relative risk (measured by means of SMR, i.e. the ratio between observed and expected cases in each area under internal standardization) was judged to be significantly greater than 1 in many municipal areas, the significance being evaluated under the null hypothesis of neutral risk on the ground of area-specific p-values (denoted by *ρ*_*i*_); in addition, it was seen that high risk areas were not randomly distributed within the province, but showed a sharp clustering. The most perceptible cluster involved a collection of municipalities around the Maglie area (Istat code: 75039), while the association among the municipalities of Otranto, Poggiardo and Santa Cesarea Terme (Istat codes: 75057, 75061, 75072) was more ambiguous. For females, it was noteworthy the significant risk excess in the city of Lecce (Istat code: 75035), where an SMR of 1.83 and *ρ*_*i *_< 0.01 have been registered. *BYM model for the province of Lecce*: For males, Bayes estimates of relative risks varied around an overall mean of 1.04 with standard deviation of 0.1, with a minimum of 0.77 and a maximum of 1.25. The posterior relative risks for females, although smoothed, showed more variation than for males, ranging form 0.74 to 1.65, around a mean of 0.90 with standard deviation 0.12. For males, 95% posterior credible intervals of relative risks included unity in every area, whereas significantly elevated risk of mortality was confirmed in the Lecce area for females (95% posterior CI: 1.33 – 2.00). *BYM model for the whole Apulia*: For males, internally standardized maps showed several high risk areas bordering the province of Lecce, belonging to the province of Brindisi, and the presence of a large high risk region, including the southern part of the province of Brindisi and the eastern and southern part of the Salento peninsula, in which an increasing trend in the north-south direction was found.

*Ecological correlation study with deprivation *(*Cadum Index*): For males, posterior mean of the ecological regression coefficient *β *resulted to be 0.04 with 95% posterior credible interval equal to (-0.01, 0.08); similarly, *β *was estimated as equal to -0.03 for females (95% posterior credible interval: -0.16, 0.10). Moreover, there was some indication of nonlinearly increasing relative risk with increasing deprivation for higher deprivation levels. For females, it was difficult to postulate the existence of any association between risk and deprivation.

*Cluster detection*: cluster detection based on a modified BYM model identified two large unexplained increased risk clusters in the central-eastern and southern part of the peninsula. Other secondary clusters, which raise several complex interpretation issues, are present.

**Conclusion:**

Our results reduce the alleged role of the industrial facilities located around the province of Taranto: in particular, air pollution produced around the city of Taranto (which lies to the west of the province of Lecce) has been often identified as the main culprit of the mortality excess, a conclusion that was further supported by a recent study on the direction of prevailing winds on Salento. This hypothesis is contradicted by the finding that those municipalities that directly border on the province of Taranto (belonging to the so-called "Jonico-Salentina" band) are those that present low mortality rates (at least for males). In the same way, the responsibilities of energy production plants located in the province of Brindisi (Brindisi province lies to the north) appear to be of little relevance. For females, given the situation observed in the city of Lecce, and given the substantial increase in mortality observed in younger age classes, further investigation is required into the role played by changes in lifestyle, including greater net propensity to smoke that women have shown since the 80s onwards (a phenomenon which could be amplified in a city traditionally cultured and modern as Lecce, as the tobacco habit is a largely cultural phenomenon). For males, the presence of high levels of deprivation throughout the eastern and southern Salento is likely to play an important role: those with lower socio-economic status smoke more, and gender differences may be explained on the basis of the fact that in less developed areas women have less habit to tobacco smoking and alcohol drinking (and other harmful lifestyles), which are seen as purely masculine behaviour: research into the role of material deprivation and individual lifestyle differences between genders should be further developed.

## Background

This study analyses the spatial distribution of mortality from lung cancer registered in the period 1992–2001 in the province of Lecce, Italy. The motivation of this work is that the Salento peninsula (traditional name of the province of Lecce, indicating the sub-region of Italy that stretches on the South of Apulia, between the Ionian Sea to the west and the Adriatic Sea to the east) represents, in Italy, a case of considerable interest: since the first report of the Epidemiological Observatory of the Region of Apulia, which examined the causes of death in a time span of five years (from 1998 to 2002), a consistent excess of lung cancer mortality among residents in the province of Lecce was found (compared to the level observed in other Apulian provinces, see [1] for data updated in 2006). We will see shortly that these results are widely confirmed by available data: it must however be pointed out that, as in previous studies, our conclusions are based on mortality rather than on incidence data (we will discuss later this key point).

The possible causes of the risk excess were the subject of intense debate (often with inadequate scientific methodology) in the local press. We can cite, for example, the alarm raised regarding emissions produced by the Enel "Federico II" power-plant of Cerano (BR), located about 30 km from the northern provincial boundary of Lecce to the north: the plant produces, according to data reported by Legambiente (data that has been given broad emphasis in local newspapers) more than one third of total national emissions of CO_2_: 14.4 million tonnes of CO_2 _per year against a national total of about 51.6 million in 2006, a figure worsened by the presence of a second power-plant operated near Brindisi by Edipower, from which a release of nearly 3.8 million tonnes of CO_2 _into the atmosphere per year has been estimated [2]. Similar accusations were raised about mega-industrial steel factories operated by the Ilva Corporation, and located in the municipalities of the city of Taranto and Statte, about 40 km from the north-western boundary of Lecce province: coke oven batteries create a carcinogenic risk for workers, because of exposure to benzene and asbestos, and given the vicinity to the city and the inadequacy of measures of pollution control, a risk also exists for the general population [3]. In both cases the transport mechanism of pollutants released into the atmosphere would be caused by the peculiar wind system in this area.

The environmental situation in the province of Lecce is characterized by the absence of apparent environmental risk sources. However, there are some noteworthy points that must be mentioned and that will be better discussed in the light of the results obtained. For example, the situation of environmental pollution is particularly alarming. Evidence from the State Forestry Department shows that in the period 2001–2002 there were 4800 illegal landfills in Italy, 600 in Apulia, and of these 50% (over 300) in the province of Lecce alone, compared to 50 in the province of Foggia and 15 in Brindisi [4]. The illegal disposal of toxic substances in the environment constitutes a risk also for agro-food and livestock activities: the illegal landfills surveyed by the State Forestry Department are in fact located in rural areas, where contact with the water springs and crops represents a serious health risk to consumers. To all this we can add, particularly in Salento, the serious problem of abandonment, or worse of illegal incineration of plastics used in agriculture, with all the well-known consequences on the food chain. There is ample confirmation of this situation contained in the report of the Environmental Protection Agency on waste disposal [5]: of more than 153,000 tonnes of special hazardous waste disposed in Apulia, only 62,000 were disposed using authorized landfills (38,000), incinerators (16,000) and treatment plants (7,000); the remaining 91,000 tons and more were disposed of illegally, usually in abandoned quarries or in warehouses rented by waste traffickers, and presumably this situation still continues. With regard to industrial structures in the area, we must mention the presence of an important plant for the cement production located since the sixties in the municipality of Galatina (Istat code: 75029), a major industrial plant in Maglie (Istat code: 75039) for olive oil refining and extraction of oil from olive residues, formerly owned by the Regional government of Apulia and rented in the early 80s by a group of local entrepreneurs: finally, it is worth noting the presence of an incinerator located in the town of Surbo (Istat code: 75083), about 20 km from Lecce, that burns hospital waste and expired pharmaceuticals.

However, it is obvious that, apart from considerations based solely on intuition or hearsay, we need a thorough descriptive epidemiological analysis, which is the fundamental tool for a better understanding of the dynamics observed in incidence/mortality rates, both in time and space; consideration of spatial dimension is always required to suggest plausible aetiological hypotheses and to identify putative sources of risk. Of course, the generation of hypotheses is only a step towards further analytical studies to confirm the suspected causal relationship (see [6-8] as useful references on the role of spatial epidemiology, and on the links with geographical information systems). Most articles appearing in the press and on specialized websites, on the other hand, report data that are too general and too aggregated in order to be considered really useful.

In an aspatial setting, traditional risk factors described in the literature include primarily the habit of smoking: [9], a classic study, estimates that about 30% of the incidence of all cancers observed in the United States during the period chosen for the survey was attributable to consumption of cigarettes. As part of the alleged causal relationship smoking-cancer, [10] shows that the most frequent locations are the oro-pharynx, the larynx, the lung, oesophagus and bladder; [11,12] report that cigarette smoking increases up to ten times the risk of occurrence of lung cancer and up to six times that of occurrence of laryngeal cancer. The relative risk remains higher than for those who never smoked, even after a period of abstinence of more than 40 years [9]. Even passive smoking is another well studied risk factor: passive smokers inhale a complex mixture of smoke and other combustion products, a phenomenon that is commonly referred to as environmental tobacco smoke (ETS). Early studies [13,14] have shown that the risk of contracting lung cancer is significantly higher in women married to smokers than women married to non-smokers, while the most recent literature is definitely focused on relations between ETS and occupational exposures such as tobacco smoke inhaled at work [15].

Another important cause of lung cancer is occupational exposure to carcinogens: the risk of lung cancer in those exposed to some dangerous substances (such as benzopyrene, asbestos or metals such as hexavalent chromium, nickel and arsenic) is on average 4–8 times greater than for the general population [16-24]. It is worth noting that environmental asbestos exposures has been repeatedly reported as a main risk factor of pleura and lung cancer incidence (in Apulia for example [25], but see also [26] for a wider review). Arsenic in drinking water is another example of environmental exposure that cannot be totally avoided [26].

The real importance of air pollution as a risk factor is still unclear: some older classic papers show that a small percentage (1–2%) of lung cancer cases can be attributed to air pollution [27,28], while the estimates of attributable risk appear decisively higher in more recent works [29]. Even the degree of urbanization and the incidence of lung cancer are sharply associated [30-32], this association could be explained by a confounding effect due to individual causes of diseases, such as smoking habits and occupational exposure, which obviously have a significantly greater importance in most densely populated areas [33]. Of course, epidemiologic evaluation has been often confounded by difficulties in defining and measuring air pollution, and evaluating the effects of low-level exposures in the general population. As we will soon see, our data show an excess of lung cancer in areas that can be regarded as weakly urbanized.

Even the role of the inert gas Radon as a risk factor is the subject of intense studies [34,35]: although chemically inert, it is also radioactive and is transformed into yet other radioactive elements, usually called "children" who are electrically charged and attach onto fine particulate matter, and can then be inhaled and deposited on the surfaces of lung tissues. Moreover, radon is a ubiquitous domestic pollutant (indoor radon) as it penetrates buildings through gas found underground [11]. We will discuss further the spatial distribution of Radon in the Apulian territory, and its possible association with disease occurrence.

In this paper we have taken into account as well the fact that socio-economic level may be a confounding variable of the area-level spatial distribution of a given disease: this is because the socio-economic variables tend to be associated with individual risk factors, while on a larger scale they are generally associated with zones of high pollution and massive presence of industrial plants [36]. So, even without a direct effect from environmental exposure, one can still detect a spurious association between putative sources of pollution and levels of incidence/mortality due to disease occurrence. The socio-economic factors can be summarized by a synthetic index of material deprivation built at the area level: such an index is usually related to the prevalence of characteristics such as unemployment, low employment rates or low-quality housing and services [37]. We will see that association with deprivation is not strong for our mortality data, but it seems to be the only reasonable hypothesis that can explain the diffuse risk increase that is almost everywhere present in the province of Lecce (except for some localized 'hot-spots' that cannot be explained on the basis of poverty level).

Given the discussion we have presented, the objective of this paper is to study on a scientifically sound basis the spatial distribution of risk for lung cancer mortality in the province of Lecce. Our goal is to demonstrate that most of the previous explanations are not supported by data, and that methods of descriptive epidemiology are of primary importance to generate sensible etiologic hypothesis. To this end, we will follow a hybrid approach that combines both frequentist and Bayesian disease mapping methods; furthermore, we define a new sequential algorithm based on a modified version of the BYM model, suitably modified to detect geographical clusters of disease and to confirm results obtained on the basis of posterior summaries of the "pure" BYM model.

## Methods

We considered the following analyses: 1) calculation of the gross incidence rate at provincial level, as well as of standardized rates to facilitate comparison with other provinces of Apulia and the comparison with the mortality rates observed in Italy and other European nations; the reference period used for these analyses differs from that used for spatial risk estimation, for the reason that we were interested to compare patterns of temporal disease evolution in the province of Lecce with respect to other Apulian provinces as well, a task that requires a larger temporal window and that was possible due to the wide availability of data at provincial level; 2) spatial analysis to build a risk map based on the specification of an area-specific Poisson model, where the high-risk areas are identified on the basis of *p*-values associated with the null hypothesis of no-increased risk; 3) spatial analysis adjusted for the presence of spatial correlation and extra-Poisson variation in area-specific relative risks, using a Besag-Yorke-Mollié (BYM) model estimated by Markov Chain Monte Carlo (MCMC) methods in Winbugs; 4) adjustment for material deprivation by inserting an ecological covariate in the BYM model to take account of socio-economic score at areal level; 5) disease cluster detection using a new model-based Bayesian paradigm based, on a suitable modification of the BYM model.

The analyses were conducted separately for both sexes: the difference in terms of incidence and/or mortality between males and females is becoming less relevant, as witnessed also by numerous references in literature. The latest data even reduce the importance of smoking as a risk factor in males, emphasizing instead the large increase in the prevalence of female smokers [38]: [39] report data about the "epidemic" of lung cancer mortality among young women in Europe. As we do not have a priori reasons to reject the hypothesis that health effects of other risk factors could be quite different between the two sexes, the adoption of separate analyses appears to be appropriate.

### Data

The data at the provincial level were obtained from the Cislaghi Italian mortality atlas based on Istat data [40], considering deaths occurred in Apulia during the period 1981–2001 for the class of disease indicated by code 162 on the ICD-9-CM classification (i.e.: malignant neoplasm of trachea, bronchus and lung, IX^th ^Revision of the International Classification of Causes of Death, published in 1979 by the World Health Organization). To analyze the overall mortality dynamics, the reference period has been divided into seven triennials: 1981–83, 1984–86, 1987–89, 1990–92, 1993–95, 1996–98, 1999–01.

To estimate the spatial distribution of mortality, the number of deaths for lung cancer in each of the 97 municipalities in the province of Lecce was considered in the period between January 1^st^, 1992 and December 31th, 2001 inclusive. The data were obtained once again from the Cislaghi atlas: the choice of an extensive reference period was due as much to the necessity to not have information too scattered within each stratum in which the dataset was divided, as the impossibility to obtain the data, because of possible identity disclosures if the reference period was too short. It should also be noted that the areas actually analyzed are 96 in total; due to the abovementioned privacy issue, the Cislaghi atlas considers the neighbouring municipalities Racale and Taviano as a single administrative unit. To draw the maps, the areas of these two municipalities were aggregated, creating a single area to which was assigned the name of the Taviano municipality. These operations were carried out on a Geographic Information System (GIS) using the union operation: the population of the new area was simply assumed as the sum of the populations of both the municipalities.

### Calculation of mortality rates for provincial data

Referring to data at provincial level, the crude mortality rate (*M*) was calculated from the following expression



where *D*_*t *_is the number of deaths observed for the cause of death considered in the period *t*, *t *+*k *(expressed in years, in our case *k *= 2 considering three years), while *R*_*t *_is the sum of the population at risk in the same period. Exploiting age-specific data in the expression of *M*_*t *_(both the numerator as well the denominator), a specific rate *M*_*t*, *j*_, for *j *= 1,..., *J*, is obtained for each age-group considered: the division originally planned from the Cislaghi atlas includes the classes 0, 1–14, 15–34, 35–54, 55–64, 65–74, 75 +, although the first two have never been taken into account, because no deaths have ever been observed in either of the triennial considered.

To make internal and international comparisons possible, eliminating the influence of age structure on mortality, we calculated the standardized rate for each triennial with the direct method applied to the standard European population [41], i.e.



where *w*_*j *_is the relative weight of the *j*-th age group in the standard European population.

### Spatial analysis 1: area-specific Poisson model

Let *Y*_*ij *_be the number of deaths observed within the *i*-th area and the *j*-th stratum in the period in question (*i *= 1,..., *N*, *j *= 1,..., *J*). As the analyses were conducted separately for males and females, the strata were constructed by classifying the cases in six broad age groups: 0–24, 25–39, 40–54, 55–69, 70–84, 85+. We can assume that, independently in each area and stratum, disease counts follow a Poisson distribution



where *p*_*ij *_is the mortality rate within the *i*-th area and *j*-th stratum, while *N*_*ij *_is the amount of person-years at risk in the time period considered (estimated with *N*_*ij *_= *k*_*y *_× *n*_*ij*_, where *k*_*y *_= 10 and *n*_*ij *_is the amount – provided by Istat – of resident population as of January 1st, 1997). Estimation of the *N *× *J *probabilities *p*_*ij *_is carried out by assuming that the proportionality relationship *p*_*ij *_= *q*_*j *_× *θ*_*i *_holds, where *q*_*j*_, *j *= 1,..., *J*, is a set of known stratum-specific reference rates, and each parameter *θ*_*i *_is an area-specific relative risk [42,43]. Indirect standardization can account for effects attributable to differences in the confounder-specific populations: it can be carried out collapsing the strata, *Y*_*i *_= ∑_*j *_*Y*_*ij*_, to obtain the following saturated Poisson model for disease counts



where *E*_*i *_= ∑_*j*_*N*_*ij*_*q*_*j *_is the number of expected deaths in the *i*-th area. The choice of the stratum-specific reference rates *q*_*j *_is crucial [44]: we estimated each rate with *q*_*j *_= ∑*Y*_*ij*_/∑_*i*_*N*_*ij *_(internal standardization, [45,46]); this approach centres the data with respect to the map, and the areas where there is an excess of risk are those in which the number of observed cases is higher than the number of expected cases.

#### P-value based maps

The obvious summaries of the Poisson model are given by the maximum likelihood estimates of each area-specific relative risk , *i *= 1,..., *N*. For the calculation of the respective confidence intervals it can be seen that , hence an estimate of the variance is given by . Assuming that  has an approximately Gaussian distribution, a first order Taylor approximation leads to ; therefore, an approximate 95% confidence interval for log(*θ*_*i*_) is given by . Back-transforming, we have the following approximate 95% confidence interval for



that may be used to summarize the significance of the statistical hypothesis of no increased risk within the *i*-th area *H*_0_: *θ*_*i *_= 1, against the alternative hypothesis *H*_0 _: *θ*_*i *_> 1 (if  > 1, otherwise the alternative hypothesis is *H*_0 _: θ _*i *_< 1 if  < 1).

The exact area-specific *p*-values associated to the null hypothesis of no increased risk *H*_0 _: *θ*_*i *_= 1 are given by



All of these values, for *i *= 1,..., *N *may be classified to draw a probability map, attributing to each area a colour level that denotes class membership [47]. At a significance level of *α *= 0.05, high-risk areas are those in which *ρ*_*i *_<*α *and  > 1 occur simultaneously.

It is worth noting that probability maps may not be very informative, as *p*-values alone do not give any information about the level of risk.

### Spatial analysis 2: the Besag-York-Molliè (BYM) model

Apart the abovementioned issues, outcomes in spatial units are often not independent of each other. Risk estimates of areas that are close to each other will tend to be positively correlated as they share a number of spatially varying characteristics. Ignoring the overdispersion caused by spatial autocorrelation in the residual leads to incorrect inferences: in particular, an extreme value of *ρ*_*i *_may be more due to the lack of fit of the saturated Poisson model than to its deviation from the null hypothesis of neutral risk. This effect of overdispersion due to spatial autocorrelation is very strong only for small area (i.e. areas with very low populations), while is negligible for large municipalities [48].

As risk estimates of areas that are close to each other will tend to be positively correlated, if all such characteristics could be properly measured, then the model would be fully specified and residual spatial variation would be fully explained. However, this will never be the case and unmeasured spatial factors will introduce spatial dependence that can be described by introducing into the model suitable random effects. This is the reason why we considered a standard BYM model for a more refined analysis [49,50]; we briefly recall the formulation of the hierarchical model:

• 1^st ^level – Disease counts are assumed to be distributed as in the saturated Poisson model, and assumed to be conditionally independent given the relative risks *θ*_*i*_;

• 2^nd ^level – The linear predictor assumes the following form



where *α *is a baseline log-relative risk, and *v*_*i *_and *ε*_*i *_are random effect representing spatial clustering (autocorrelation) and unstructured extra-Poisson variation respectively. Distributional forms commonly adopted at the second level for the two random effects are:

**Clustering **– *v*_*i *_is defined by the CAR (Conditional Autoregressive, [51]) specification , where , ∂_*i *_is the set of all areas neighbouring the *i*-th area, and *n*_*i *_is the number of elements within the set. Hence, the CAR effect describes spatially varying risk factors, based on which neighbouring areas tend to have similar relative risks. The specification of a CAR effect forces the estimates of area-specific log-relative risks toward a local mean (with an obvious smoothing effect and noise reduction of the map);

**Heterogeneity **– *ε*_*i *_is used instead to describe the sources of error not spatially structured: for this reason we hypothesize, as usual, the exchangeable specification .

• 3^rd ^level – Prior distributions of the parameters of the two random effects must be chosen [52]: let *G*(*a, b*) denote the Gamma distribution with expected value *a*/*b *and variance *a*/*b*^2^. For each of the two precision parameters,  and , we set *τ*_*v *_~ Gamma(*a*_*v*_, *b*_*v*_) and *τ*_*ε *_~ Gamma(*a*_*ε*_, *b*_*ε*)_. In this paper we used *a*_*v *_= 0.5, *b*_*v *_= 0.005 for the spatially structured component [53], which corresponds to a diffuse prior that does not artificially force a spatial structure in the log-relative risk estimates when this is not actually present in the data. By simple calculations it can be proven that, with this choice, the standard deviation of spatially structured random effects is a random variable centred around 0.01, and the probability of observing values smaller than 0.01 or larger than 2.5 is equal to 0.01. For the non-spatially structured random effect we set *a*_*ε *_= 0.01, *b*_*ε *_= 0.01: these values correspond to a non-informative prior, and they were chosen on the ground of an empirical trade-off between the need to not use an overly informative prior (given the previous absence of knowledge on spatial distribution of mortality from lung cancer), and the care taken to avoid deterioration of the convergence of the estimation algorithm, a very common issue when a flat prior is used.

Prior to model estimation we tested SMRs for the presence of overdispersion and spatial autocorrelation, as the use of BYM model needs to be motivated if the evidence for spatial autocorrelation or extra-Poisson variation is not strong. We applied the following battery of tests: Pearson chi-square and Potthoff-Whittinghill's statistics for assessing homogeneity of relative risks [54]; Dean's overdispersion score for testing the presence of extra-Poisson variation versus the null hypothesis of Poisson distribution [55]; Moran's *I *and Geary's *C *tests to assess the presence of spatial autocorrelation, accounting for over-dispersion by assuming a negative-binomial distribution as the sampling model needed to compute the null distribution by means of parametric bootstrap [56].

#### Parameter estimation and disease mapping

Posterior estimates of the parameters were obtained by simulating from the joint posterior by means of a Markov Chain Monte Carlo (MCMC) algorithm, using the OpenBugs 3.0.3 software together with the GeoBugs 1.3 extension [57]; 6,000 burn-in iterations on two parallel chains starting from overdispersed values were simulated: the convergence was checked using the Brooks-Gelman-Rubin diagnostic, summarized with the  coefficient which tends to 1 in case the convergence is achieved [58]. Subsequently, another 3,000 iterations (for each chain) were generated: only one out of each three was considered for estimation, in order to eliminate the serial autocorrelation and to reduce the standard error estimate of the parameters. We monitored and estimated the area-specific relative risks *θ*_*i *_by means of the MCMC algorithm described in the previous section. Maps were drawn by dividing the whole range of relative risk posterior estimates in five non-overlapping sub-intervals delimited by quintiles, assigning a suitable colour to each interval and classifying areas accordingly.

Within the context of cluster detection, questions have been raised about the performance of the BYM model in recovering the true risk surface. For this reason, rather than insisting on the interpretation of relative risk posterior estimates, we supplemented our result by monitoring and estimating area-specific posterior probabilities *δ*_*i *_= *E*[*I*(*θ*_*i *_> 1)|*Y*] = Pr{*θ*_*i *_> 1|*Y*} (here *I*(•) denotes the event indicator function). Based on those new posterior area-specific summaries, maps were drawn dividing the interval [0,1] in ten equally spaced sub-intervals, and assigning a colour to each area accordingly. The resulting maps are likely to be insufficiently informative on the actual risk level (as well as p-value based maps are), but the may be indeed useful to confirm the presence of "hot-spots" of high-risk areas exploiting results given in a wide simulation experiment based on synthetic data, where a feasible benchmark for the risk calibration problem was provided [59]. The authors, defining three different loss function representing weighted tradeoffs between false positive and false negative, showed that by declaring at "high risk" those areas where  > 0.8, each area with relative risk below 2 was classified as high risk with a probability of at least 75% if the expected cases in each area are between 10 and 20; this probability approximates to 1 for areas with a relative risk of 3 if the expected cases are never less than 5.

#### Correction for edge effects

Even when disease counts are independent, any smoothing operation applied to SMRs that borrows information from neighbouring areas will induce edge effects, that consists in biased estimates in areas located near the boundary of the investigated region because information on what happen on the other side of the boundary is missing: a larger estimation variance will be also found in boundary areas due to the low proportion of neighbouring cases [60]. Because some putative sources of pollution are located outside the study area, and the estimation of large-scale patterns are likely to be affected by such statistical biases, it was necessary to adjust for edge effects. A classical methods is to employ guard areas, which are areas external to the main study window of interest and are added to the window to provide a guard area [60]: in this case, given the availability of mortality data for the whole Apulia, it was possible to estimate a global disease map in order to assess, without distortions, the presence of spatial patterns originating from the putative sources located around Brindisi and Taranto, and the large-scale structure of disease risk. In order to make comparisons between the two maps feasible, the stratum-specific reference rates *q*_*j *_for the Apulia map were set equal to those used for the Lecce map (*external standardization*): in this way, expected counts for areas located inside the province of Lecce resulted to be the same for both maps. We also considered internal standardization for the Apulia map; for the province of Lecce considered in isolation, this is essentially equivalent to shift upward posterior estimates obtained by external standardization, but it may be undoubtedly useful in order to compare the average disease risk level in the province of Lecce with that occurring in the whole Apulia. Spatial risk estimation was carried out by simulating 12,000 MCMC burn-in iterations, and subsequently another 6,000 iterations (for each chain) considering only one out of each three for estimation.

### Spatial analysis 3: accounting for socio-economic factors

As we said, for many diseases there is a strong link between health and material deprivation, and pollution sources tend to be found in deprived areas. Hence, deprivation is a potential confounder, the role of which must be carefully examined during the analysis. This is the reason why we considered an ecological correlation model as well, in which the linear predictor of the BYM model is expanded in the following way



The area-specific deprivation measure *x*_*i *_considered here is the Cadum index [61]: this measure is well suited to the information flows available in Italy, and was calculated using Census data provided by Istat for the 1991 census. The amounts taken into consideration in constructing the index are as follows:

• proportion of population with primary education;

• proportion of rental housing;

• proportion of occupied residences without bathroom;

• proportion of the active workforce unemployed or looking for a first job;

• proportion of single-parent families with children.

The index in question is constructed by calculating, for each area, the *z *score of each variable standardized with respect to the national average and to the national standard deviation, and then adding the five scores thus obtained: by design, higher scores are observed in poorer areas. A posterior summary of the importance of Cadum index in explaining the spatial pattern of disease risk can be obtained by monitoring and drawing residual spatial variation exp(*v*_*i *_+ *ε*_*i*_), an area-specific quantity that is adjusted to eliminate the net effect due to the level of material deprivation in the *i*-th area: the presence of a weak spatial pattern in residual spatial variation indicates a strong association with deprivation. Alternatively, the ecological coefficient *β *can be considered significantly positive if its posterior mean is greater then zero and its 95% percent credible interval excludes zero: this means that there is an actual risk increase in those areas where the level of poverty is higher. It is also interesting to note that when a covariate inserted in an ecological BYM model result to be appropriate to model the spatial variation of risk, random effects *v*_*i *_and *ε*_*i *_may become unidentifiable and care must be taken to avoid poor convergence and invalid inferences if an improper posterior is used [62].

### Spatial analysis 4: cluster detection and inference

As we said, several authors noted that the smoothing effect of the BYM model results in a correct estimation of the spatial distribution of disease risk, but it renders almost impossible the detection of localized increases if these are not based on large local excesses [59,63]. It may be the case that 95% relative risk credible intervals include unity in all areas, even when local risk excesses are actually present in the data. For this reason we considered a version of the BYM model, suitably modified to detect geographical clusters of disease and to confirm results obtained interpreting posterior summaries of the "pure" BYM model: if Δ is a collection of candidate zones, i.e. the set full set of possible clusters given a maximal dimension, conditionally to each cluster *Z *∈ Δ the linear predictor is reformulated as



where a cluster-specific effect *α*_*Z *_enters the model as the coefficient of the dummy variable *I*(*A*_*i *_∈ *Z*), which assumes value 1 if area *A*_*i *_is in *Z *and 0 otherwise. In a like vein to the model-based approach introduced in [64], where an overdispersion parameter similar to the general overdispersion parameter in Poisson regression was used to reduce the effect of extra-Poisson variability on cluster location detection, our Bayesian formulation expressly addresses the problem that cluster detection nominal type I error and power of classical Scan Statistics are likely to be appreciably affected by the presence of spatial autocorrelation [65]. We propose the following sequential model-based algorithm for cluster detection (here by "cluster" we mean any collection of neighbouring areas):

• Given an initial collection Δ of candidate zones, conditionally to *Z*, we fit our model for all *Z *∈ Δ by suitably tuned MCMC simulations. The collection of fitted models is ranked (in terms of parsimony and predictive power) on the ground of the Deviance Information Criterion (DIC, which is a hierarchical modelling generalization of Akaike Information Criterion introduced in [66]: between two competing models the one that has a lower DIC score should be preferred, see the legend of Tab. 3 for further details), and the first cluster Z* is identified accordingly, by means of the cluster-specific indicator variable entering the model that has the lowest DIC value; a non-informative Gaussian prior is usually assumed for *α*_*Z*_;

• Let  be posterior estimate of the cluster-specific effect: a new model containing an additional offset term accounting for the effect due to Z* is considered, treating *I*(*A*_*i *_∈ Z*) as an explanatory variable with known coefficient equal to . Let Δ* be the collection of zones including Z* and every cluster overlapping with Z*: the previous step is iterated assuming Δ - Δ* as the collection of candidate clusters, and a second optimal cluster is identified in case;

• The procedure stops when no better data explanation is possible by letting further cluster-specific terms enter the model: this is easily appreciated by means of the sequence of the DIC scores of the best model of each iteration, in the sense that the algorithm ends when such sequence becomes increasing.

It should be noted that cluster location detection and cluster inference are quite distinct: the above-described algorithm reduces the number of feasible clusters by comparing a large number of models on the ground of a data-analytic criterion. Anyway, declaring a cluster *Z *statistically "significant" is a different task: in our Bayesian approach this occurs when 95% posterior credible interval for the cluster-specific log-relative risk *α*_*Z *_excludes zero.

## Results

**Figure 1:**
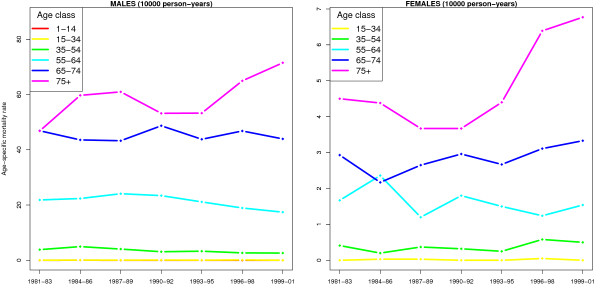
**Age-specific lung cancer mortality rates in the province of Lecce – reported by triennial, covering the period 1981–2001**. The age groups used correspond to those given by the output of the Cislaghi mortality atlas. The age groups in which no deaths were observed in each of the triennial considered were not reported (e.g. age class 1–14 for females). For males, lines corresponding to age classes 1–14 and 15–34 are indistinguishable.

A first idea on the size of the phenomenon can be obtained by studying the specific mortality rates calculated by sex and age, as reported in Figure 1; at a first glance, a general increase in mortality over time would not seem obvious if the 75 + class is excluded, for which, analyzing males, there is a shift from 53.28 deaths per 10,000 person-years in the triennial 1993–95 to 71.50 deaths per 10,000 person-years per year in the triennial 1999–01 (with a percentage increase of 34.2%); for females, referring to the same triennials, the rates rose from 4.40 to 6.77 deaths per 10,000 person-years, an increase of 53.86%. Such a sharp rise in mortality may be explained by the increase of average life expectancy of individuals belonging to this age class, since the cumulative probability of contracting the disease as a result of multiple exposures to risk factors increases with age. However, it must be noted that for females there is an evident increase in other younger age groups as well, for example 35–54, where 0.25 deaths per 10,000 person-years was recorded in the triennial 1993–95, compared to 0.50 deaths in the triennial 1999–01, with a percentage increase of 100%: this dynamic of strong growth is confirmed in many other studies and will play an important role in interpreting the phenomenon.

**Figure 2:**
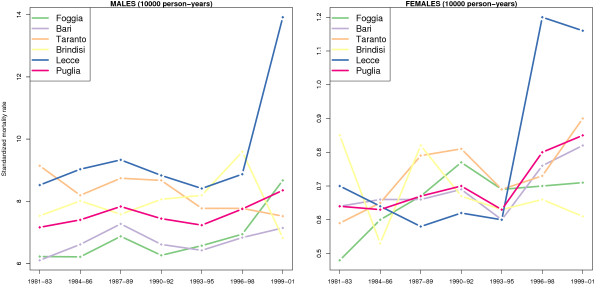
**Standardized lung cancer mortality rates for the five provinces of Apulia and for Apulia as a whole – reported per triennial – covering the period 1981–2001**. The direct method applied to the European standard population was used for the standardization.

In Figure 2 we have reported the standardized mortality rates for the five Apulian provinces and for Apulia as a whole, using the European standard population as a reference. The province of Lecce, at least for males, shows rates significantly above the Apulian average for all seven triennials considered (a behaviour that is confirmed in the provinces of Brindisi and Taranto, albeit with consistently lower amounts): the standardized rate increases from 8.41 (per 10,000 person-years) in the period 1993–95, to 13.92 for the triennial 1999–01 (percentage increase: 93.33%). It is obvious that this dramatic increase is the biggest problem in terms of public health management.

The alarming extent of the phenomenon appears even more evident if, considering the triennial 1999–01, national and international comparisons are made with suitable data. For example, the AIRT 2006 report on cancer in Italy displays for males a standardized rate for Italy equal to 6.96 per 10,000 person years in the triennial 2000–02, while for females the corresponding rate is 1.27 per 10,000 person years [67] (rates of the AIRT study were calculated by means of standardization with respect to European standard). The same study shows, for Apulia, 6.64 deaths for males and 0.73 for females (per 10,000 person-years, triennial 2000–02): these figures are unquestionably comparable to those we calculated for the period 1999–01 for Apulia (8.35 for males and 0.85 for females). Therefore, with reference to the period 1999–01, we can conclude that death due to lung cancer in the province of Lecce is almost double for males, compared with the national and Apulian average, while for females it is much higher than the Apulian average but comparable to the national average; once again there is a disparity between the two sexes that, as we will soon see, will be confirmed in the geographical analysis.

**Figure 3:**
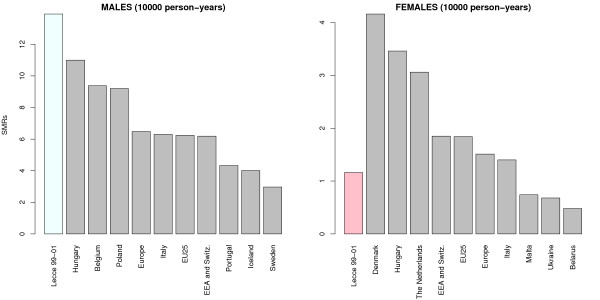
**Standardized lung cancer mortality rates for the province of Lecce in the triennial 1991–01, compared with the standardized rates of some European aggregates**. The direct method applied to the European standard population was used for the standardization. Countries that appear in this graph are, respectively, those who have registered the three highest and the three lowest standardized rates in 2006.

Some international comparisons have been highlighted in Figure 3, based on [68], which contains data for 2006: in Italy there were 6.3 deaths per 10,000 person-years for male, while deaths among females were 1.4 per 10,000 person-years. The only European nation that has standardized rates at the level of the province of Lecce is Hungary, with 11 deaths per 10,000 person-years for males. Slightly different is the situation for females, since many European countries have very high mortality rates, well above the European average and the level registered in the province of Lecce. In some countries, like Denmark, the female mortality rate is only slightly less than that observed for males.

### Spatial analysis: results

#### SMRs for lung cancer in the province of Lecce

**Figure 4:**
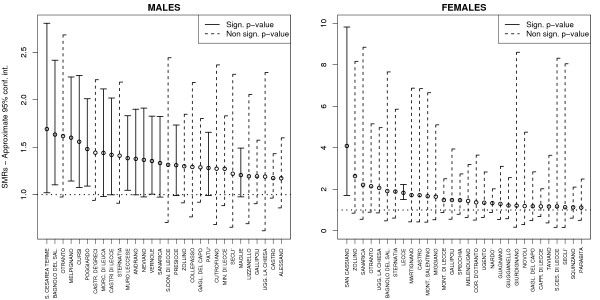
**Standardized Mortality Ratios (SMRs) and their 95% approximate confidence intervals (lung cancer mortality in the province of Lecce, 1992–2001)**. All areas have been ordered in a descending order based on the SMR value, and of these only the first 30 were considered for the graphic representation. Statistical significance refers to the null hypothesis of no increased risk *H*_0 _: *θ*_*i *_= 1, where the alternative hypothesis is Ho: *θ*_*i *_> *1*. The horizontal dashed line indicates the no increased risk line: it is worth noting that due to the approximation used for the construction of confidence intervals, the lower end may fall below that line (although a few decimal places only) even when significance is reached.

**Figure 5:**
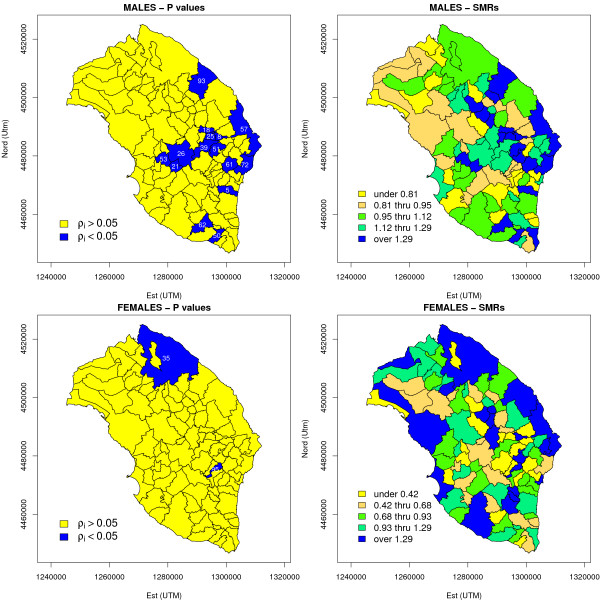
**Risk maps based on the p-value of the null hypothesis *H*_0 _: *θ*_*i *_= 1 (lung cancer mortality in the province of Lecce, 1992–2001)**. Only areas in which *θ*_*i *_> 1 and *ρ*_*i *_< 0.05 were highlighted, i.e. those areas where the risk excess resulted to be statistically significant. The numbers reported are the last two digits of the Istat municipal code.

the Istat municipal codes of the areas considered in this paper are listed in the Additional File 1 in order to enable their identification in the maps. Table 1 presents the estimated SMRs for selected areas, ordered by decreasing SMR and reporting only those areas where *θ*_*i *_> 1 and *ρ*_*i *_< 0.05 simultaneously occurred (i.e. the risk excess resulted to be significant according to the area-specific Poisson model). For males, SMRs varied around their overall mean 1.04 with standard deviation 0.28, ranging from 0.11 to 1.69; for females the variation was far more extreme, with an overall mean of 0.88 and standard deviation of 0.66, and values ranging from 0 to 4.09. For each SMR we also computed 95% approximate confidence intervals: those excluding unity, and thus regarded as statistically significant are presented in Figure 4. It is worth noting that due to the approximation used for the construction of confidence intervals, the lower end may fall below the neutral risk line *θ *= 1 (although a few decimal places only) even when significance is reached: for females, the standard errors for SMR are quite higher. Examining Table 1, it is worth noting the significant risk excess for females in the city of Lecce (Istat code: 75035), where an SMR of 1.83 has been registered, and for the area of San Cassiano (Istat code: 75095) where the correspondent SMR is 4.09 and *ρ*_*i *_< 0.01 (it is clearly seen that confidence intervals do not include unity). Looking instead at the results for males, it must be highlighted that many municipal areas reach the significance level; in addition, by looking at Figure 5, it is seen that high risk areas are not randomly distributed within the province, but show a sharp clustering. The most perceptible cluster involves a collection of municipalities around the Maglie area (Istat code: 75039), while the association among the municipalities of Otranto, Poggiardo and Santa Cesarea Terme (Istat codes: 75057, 75061, 75072) is more ambiguous. As we said, these results must be confirmed by more specialized methods, as maps based on SMRs are likely to be affected by random Poisson noise and extra-Poisson variation, whereas p-value based maps are insufficiently informative on the actual level of risk.

**Table 1:** Maximum likelihood (SMR) and Bayesian estimates of the relative risk of mortality for lung cancer in the province of Lecce, 1992–2001, for selected areas

*Istat Code*	*Area*	*Y*_*i*_	*E*_*i*_	*SMR*	*Significance*	*95% CI*_*SMR*_		*95% CI*_*Bayes*_
**Males**								
75008	Bagnolo del Salento	15	8.87	1.69	0.05	(1.02–1.81)	1.21	(0.92–1.56)
75072	Santa Cesarea Terme	25	15.31	1.63	0.02	(1.10–2.42)	1.23	(0.96–1.55)
75057	Otranto	34	21.24	1.60	0.01	(1.14–2.24)	1.25	(0.99–1.57)
75025	Cursi	28	17.97	1.56	0.02	(1.08–2.26)	1.26	(0.98–1.64)
75061	Poggiardo	41	27.69	1.48	0.02	(1.09–2.01)	1.20	(0.96–1.47)
75018	Castrignano de'Greci	26	18.06	1.44	0.05	(0.98–2.11)	1.20	(0.95–1.50)
75050	Morciano di Leuca	31	21.84	1.42	0.04	(1.00–2.02)	1.17	(0.92–1.51)
75093	Vernole	49	35.39	1.38	0.02	(1.05–1.83)	1.18	(0.95–1.46)
75051	Muro Leccese	37	26.87	1.38	0.04	(1.00–1.94)	1.19	(0.95–1.49)
75005	Andrano	34	24.88	1.37	0.05	(0.98–1.91)	1.12	(0.90–1.41)
75053	Neviano	43	31.73	1.36	0.04	(1.00–1.83)	1.15	(0.92–1.40)
75064	Presicce	39	29.25	1.33	0.05	(0.97–1.82)	1.13	(0.91–1.41)
75021	Collepasso	49	37.39	1.31	0.04	(0.99–1.73)	1.14	(0.93–1.38)
75026	Cutrofiano	58	45.27	1.28	0.04	(0.99–1.66)	1.16	(0.95–1.39)
75039	Maglie	86	71.28	1.21	0.05	(0.98–1.49)	1.16	(0.98–1.37)

**Females**								
75095	San Cassiano	5	1.22	4.09	0.01	(1.70–9.82)	1.20	(0.69–2.20)
75035	Lecce	100	54.62	1.83	0.01	(1.50–2.23)	1.65	(1.33–2.00)

Estimates are shown ordered by decreasing SMR. For males  is the posterior mean relative risk estimated using the fully BYM model with clustering and heterogeneity random effects; for females, only the heterogeneity term was considered. The column '*Significance*' must be read as "p-value *ρ*_*i *_of the null hypothesis of no increased risk *H*_0 _: *θ*_*i *_= 1 is strictly less than the indicated value". The areas reported in the table are those where *θ*_*i *_> 1 and *ρ*_*i *_< 0.05, i.e. the risk excess resulted to be statistically significant. It is worth noting that due to the approximation used for the construction of confidence intervals, the lower end of *95% CI*_*SMR *_may fall below unity (although a few decimal places only) even when significance is reached.

#### BYM model for the province of Lecce

prior to model estimation, we tested the SMRs for overdispersion and spatial autocorrelation. Results are presented in Table 2: for males, the presence of both sources of extra-Poisson variation was widely supported, and spatial autocorrelation was still found even after accounting for over-dispersion (p-values for Geary's *C *and Moran's *I *were respectively 0.03 and 0.01, assuming a Negative Binomial as the null sampling distribution): this is in agreement with the strong spatial pattern present in relative risk estimates. For females, the situation is less structured; the absence of a quite clear spatial pattern was reflected into the fact that spatial autocorrelation tests resulted to be not significant (p-values are 0.53 and 0.64), and that most of the deviation from the null hypothesis that relative risks are distributed according to a Poisson random variable could be explained by the risk excess in the Lecce area. In fact, we carried out tests twice, respectively including and excluding the Lecce area, and when Lecce was not included even the chi-square homogeneity test resulted to be not significant (p-value 0.42). For these reasons, for females, the random-effect model used for Bayes relative risk estimation did not include the clustering term.

**Table 2:** Tests for assessing the presence of heterogeneity and spatial autocorrelation in the relative risks of mortality for lung cancer in the province of Lecce, 1992–2001

*Test*	*Sampling null distribution*	*Number of iterations*	*P-value*
**Males**			
Pearson chi-squared	Multinomial	9999	0.01
Pothoff-Whittinghill	Multinomial	9999	0.01
Dean's over-dispersion test			0.01
Geary's *C*	Negative Binomial	9999	0.03
Moran's *I*	Negative Binomial	9999	0.01
**Females**			
Pearson chi-squared	Multinomial	9999	0.02
Pearson chi-squared *	Multinomial	9999	0.42
Pothoff-Whittinghill	Multinomial	9999	0.01
Pothoff-Whittinghill *	Multinomial	9999	0.42
Dean's over-dispersion test			0.01
Dean's over-dispersion test*			1.00
Geary's *C*	Negative Binomial	9999	0.53
Moran's *I*	Negative Binomial	9999	0.64

Parametric bootstrap under used, when necessary, to compute the significance of the observed values. For Geary's *C *and Moran's *I *a Negative Binomial bootstrap sampling distribution under the null hypothesis was used, in order to account for the presence of extra-Poisson variation. For females, some tests were conducted twice: the star '*' means that the test was carried out excluding the Lecce area.

**Figure 6:**
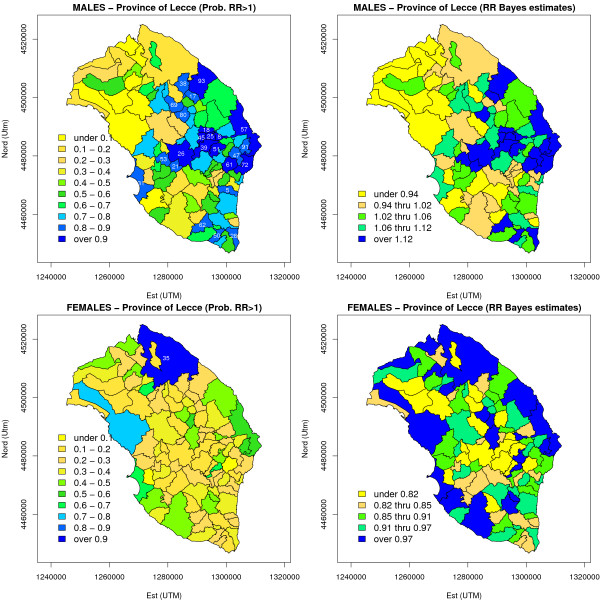
**Risk maps based on BYM model (lung cancer mortality in the province of Lecce, 1992–2001)**. MCMC estimates of posterior probability *δ*_*i *_= Pr{*θ*_*i *_> 1|*Y*} and relative risk *θ*_*i *_were reported for each area: for *δ*_*i *_a scale of 10 equidistant intervals was used, at variance with relative risks for which quintile based maps were drawn. The areas in which  > 0.8 were highlighted, showing the last two digits of the Istat municipal code. See the legend of Table 2 for further details about Bayesian models used for relative risk estimation.

The shrinkage effect was quite strong for both sexes (for females, the information is borrowed by means of the common variance of the heterogeneity effect): for males, Bayes estimates of relative risks varied around an overall mean of 1.04 with standard deviation of 0.1, with a minimum of 0.77 and a maximum of 1.25. For females, the posterior relative risk surface, although smoothed, showed more variation than males, ranging form 0.74 to 1.65, around a mean of 0.90 with standard deviation 0.12. Selected 95% posterior credible intervals are presented in Table 1: they included unity in every area for males, whereas significantly elevated risk of mortality was confirmed in the Lecce area for females (95% posterior CI: 1.33 – 2.00). Most notably, the San Cassiano area resulted to be not significant (95% posterior CI: 0.69 – 2.20). Maps presented in Figure 6 confirm the different spatial patterns for the two sexes: the spatial distribution of areas in which  > 0.8 provide further insights about the detection of high-risk areas. For males the presence of two large, apparently separate, clusters is now even more obvious in central-eastern Salento around the Maglie and Otranto areas respectively, and another grouping formed by some municipalities close to Lecce and grouped around Vernole (Istat code: 75093), which was the only one of that group to present a significant excess of risk even on p-values based maps (Figure 5 and Table 1). This "highlighting" effect is due to the use of spatially structured random component which, as we know, tends to adjust the relative risks towards a local average and pushes up the risk of those areas closer to significance and bordering an area in which the relative risk is already significantly higher than 1. Other isolated high risk areas emerge in the south of the peninsula, towards the cape, such as Morciano di Leuca (Istat code: 75050) showed a significant p-value as well (Table 1): in these cases, it is obviously much more difficult to identify putative risk factors, which in this case should carry on their effects on a very short scale. For females on the other hand, the role played by Lecce is clearly confirmed.

#### BYM model for the whole Apulia

**Figure 7:**
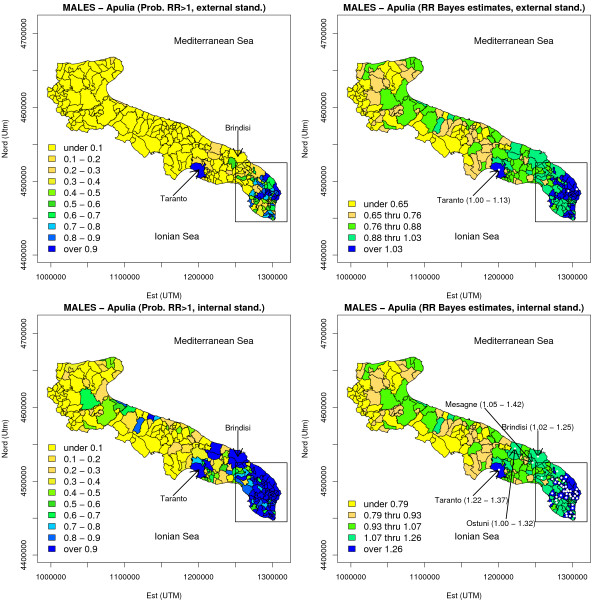
**Risk maps for the whole Apulia based on BYM model (MALES, lung cancer mortality, 1992–2001)**. MCMC estimates of posterior probability *δ*_*i *_= Pr{*θ*_*i *_> 1|*Y*} and relative risk *θ*_*i *_were reported for each area: for *δ*_*i *_a scale of 10 equidistant intervals was used, at variance with relative risks for which quintile based maps were drawn. A fully Bayesian BYM model was used for relative risk estimation. The geographical position of the province of Lecce was highlighted by means of a rectangular box. Areas in which  > 1 and the corresponding Bayesian 95% credible intervals for relative risks excluded unity were highlighted by means of a dot; areas located outside the province of Lecce, and in which the corresponding relative risk resulted to be significant in the above defined sense, were explicitly marked by an arrow.

**Figure 8:**
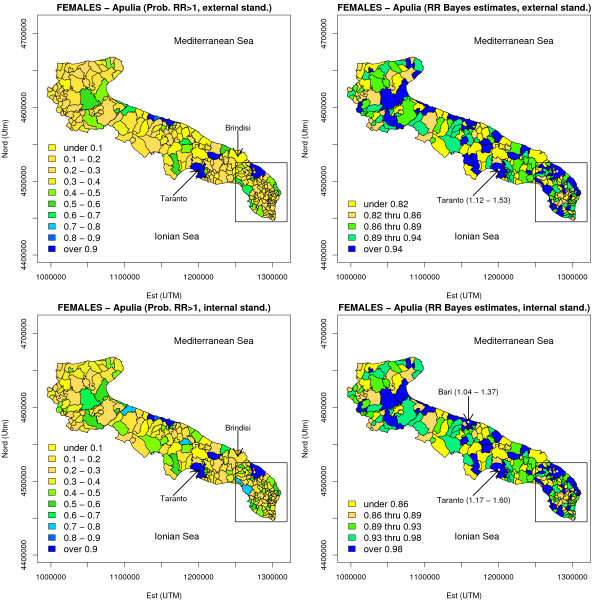
**Risk maps for the whole Apulia based on the BYM model (FEMALES, lung cancer mortality, 1992–2001)**. See the legend of Figure 7. A Bayesian specification with the only heterogeneity random effect entering the model was used for relative risk estimation.

the analysis of spatial variation of risk on a larger scale (considering the whole Apulia) is presented in Figures 7 and 8. Externally standardized maps (Figure 8) show that relevance of edge effects is clearly limited: it is worth noting that risk estimates in the Taranto area, for which we have reminded the presence of an high environmental risk, are strongly significant both for males and females. Internally standardized maps invite for several remarkable considerations. For males, several areas bordering the province of Lecce, and belonging to the province of Brindisi, were found to be at high risk: we may indeed conclude that there is a large high risk region (known as 'Big Salento'), including the southern part of the province of Brindisi, and the eastern and southern part of the Salento peninsula, in which an increasing trend in the north-south direction is clearly seen. It is noteworthy that the municipalities located within the "Jonico-Salentina" belt (i.e. bordering the province of Taranto) are those with the lowest overall mortality rates (at least for males), and no apparent spatial pattern ore trend toward the Taranto area are present. For females, the localized elevation of risk in the Lecce area is once again manifest.

#### Ecological correlation study with deprivation (Cadum index)

**Figure 9:**
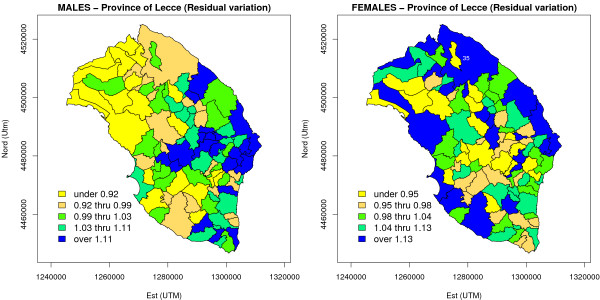
**Residual spatial variation after adjusting the BYM model for an ecological covariate, the Cadum deprivation index (lung cancer mortality in the province of Lecce, 1992–2001)**. For males, MCMC estimate of residual spatial variation exp(*v*_*i *_+ *ε*_*i*_) was reported for each area using a quintile based maps, in order to assess the presumptive effect due to the level of material deprivation in the *i*-th area. For females, the residual spatial variation considered to draw the map was exp(*ε*_*i*_), as the clustering random effect did not enter the Bayesian model used for relative risk estimation.

**Figure 10:**
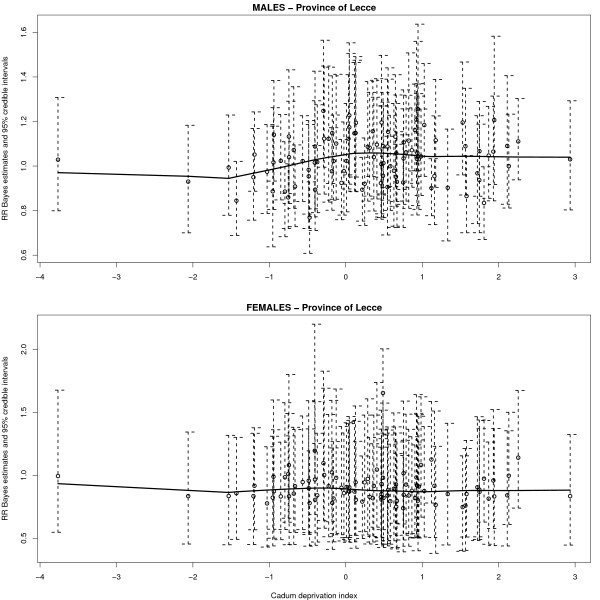
**Relative risk Bayes estimates with 95% credible intervals plotted versus Cadum deprivation score, with local smoother superimposed (lung cancer mortality in the province of Lecce, 1992–2001)**. See the legend of Table 2 for further details about Bayesian models used for relative risk estimation.

the Cadum index for the province of Lecce varied around an overall mean of 0.31 with standard deviation of 1.03, ranging from -3.80 to 2.90 (the third quartile being equal to 0.93). It is quite interesting to note that the distribution of the Cadum index for Italy assumes values between -5.42 and 34.40, and that 99% of the distribution falls within the interval (-3.4, 6.24), excluding more extreme values ([61], based on 1991 Italian census data). Therefore, it can be concluded that the entire province of Lecce is an area presenting a high level of deprivation. The results based on the BYM model in which the Cadum index was introduced as ecological covariate are shown in Figure 9, in which residual spatial variation is presented. Due to convergence problems, the MCMC run required 60,000 MCMC burn-in iterations, and subsequently another 30,000 iterations (for each chain) considering only one out of each three for estimation. For males, posterior mean of the ecological regression coefficient *β *resulted to be 0.04 with 95% posterior credible interval equal to (-0.01, 0.08); similarly, *β *was estimated as equal to -0.03 for females (95% posterior credible interval: -0.16, 0.10). Moreover, it is clearly seen from Figure 9 that the spatial structure of the unexplained variation is very similar (especially for men) to the spatial pattern shown by Bayes estimates of relative risk presented in Figure 5: it seems that these results exclude the existence of a positive linear association between risk and deprivation. The real picture is however slightly more complex: Figure 10 plots Bayes estimates of relative risks (Figure 5) versus Cadum index; each estimate has an associated 95% posterior credible interval, and a local scatterplot smoother was superimposed. We see that, for males, there is some indication of nonlinearly increasing relative risk with increasing deprivation for higher deprivation levels: we know from previous analyses that areas with the highest relative risks are located in the eastern and southern part of the Salento peninsula, and it is precisely in those areas that the highest scores of the Cadum index occur. For females, according to Figure 10, it is actually difficult to postulate the existence of any association between risk and deprivation.

#### Cluster detection

**Figure 11:**
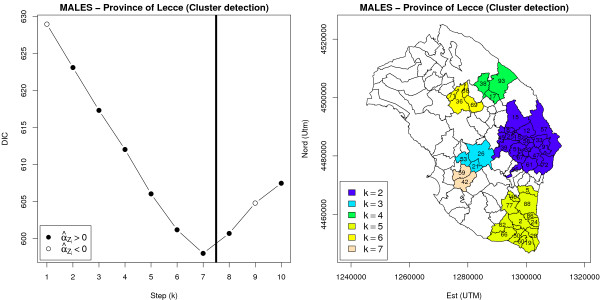
**Cluster detection with a modified version of the BYM model (MALES, lung cancer mortality in the province of Lecce, 1992–2001)**. On the left the first ten steps of sequential cluster detection algorithm were reported: the vertical solid line corresponds to the turning point of DIC score sequence. On the right, detected clusters were highlighted. The initial set Δ was defined as the set of all circular neighbourhoods using 10% of total expected counts to define the maximum circle size.

for cluster detection based on the modified BYM model, the scanning procedure considered the set of all circular neighbourhoods, using 10% of total expected counts to define the maximum circle size. Results for males are presented in Figure 11 and Table 3: our approach strengthened the evidence for the presence of two large unexplained clusters in the central-eastern and southern part of the peninsula. To check the sensitivity of these results with respect to the choice of Δ, we repeated the analysis using 50% of total expected counts to define the maximum circle size: identified patterns were found to be virtually identical, except that clusters respectively estimated during steps *k *= 3,7 and *k *= 4,6 were merged into a single cluster (results are available from the authors upon request). Once again it is apparent the presence of unexplained high-risk zones, that the BYM model was not able to highlight.

**Table 3:** Results of the cluster detection sequential algorithm based on a suitable modification of the BYM model.

**Step (*k*)**	#(Δ -Δ*)		*95% CI*_*Bayes*_		*P*_*D*_	*DIC*
**2**	547	0.25	(0.12, 0.39)	591.44	31.67	623.10
**3**	341	0.29	(0.08, 0.50)	589.61	27.71	617.32
**4**	298	0.31	(0.08, 0.53)	578.76	24.27	612.03
**5**	249	0.18	(0.05, 0.32)	584.91	21.13	606.05
**6**	141	0.23	(0.03, 0.42)	581.26	19.93	601.20
**7**	102	0.32	(0.02, 0.62)	577.96	20.06	598.02

The DIC criterion is defined as DIC =  + *p*_*D*_, where  is the posterior expected value of deviance (which is a measure of the goodness of fit of the model), while *p*_*D *_is a penalty term, i.e. the effective number of parameters given the complex interdependencies that are introduced into the actual number of parameters by the specification of correlated random effects for the risk distribution. Between two competing models the one that has a lower score of the DIC criterion should be preferred. Here, #(Δ - Δ*) denotes the number of elements in the current collection of candidate clusters: the initial set Δ was defined as the set of all circular neighbourhoods using 10% of total expected counts to define the maximum circle size. Only significant clusters for which  > 0 were reported.

## Discussion

The results of this work, reported in the previous paragraph, makes it appear that lung cancer mortality in the province of Lecce is assuming alarming dimensions. Within the province itself, however, situation differs from municipality to municipality, but it is unquestionably not homogeneous between males and females, and this raises the problem of a rather complex interpretation. For males, as previously mentioned, the presence of two large clusters is apparent, one group of the municipalities gathered around the Maglie (Istat code: 75039) and the other located in the south of peninsula; for females, however, higher mortality rates are seen in the municipality of Lecce. Furthermore, the temporal dimension must not be neglected: Figure 2 shows clearly how, for males, the problem of increased incidence of lung cancer has subsisted, according to data available to us, for at least 25 years. Data on smoking habits and other lifestyle choices, disaggregated at the municipal level, are not available: recent data on the entire region (which unfortunately are not available separately for the two sexes) show that the percentage of non-smokers is higher than the Italian average (64% versus 56%); they show, instead, a percentage of overweight people higher than the Italian average (36.6 against 33.9), but in any case aligned with the other regions of South Italy [69].

With regard to individual risk factors, particular attention was given, in the past, to the distribution of Radon in the Apulian territory. In a major study conducted on a sample of 310 residences in nine municipalities of Apulia (Bari, Rutigliano, Foggia, Troia, Sant'Agata di Apulia, Taranto, Latiano, Lecce e Castri di Lecce) changes in the concentration of indoor Radon in these homes were assessed during the spring-summer and autumn-winter periods, and considered in association with the architectural configuration of the building and to construction materials used [70]. The reported results demonstrated that in the two selected municipalities of Lecce (Lecce and Castri di Lecce) the concentration of Radon was clearly greater than that of the other municipalities studied. The causes of this excess in these homes are mainly attributable to two factors: the building type and the geological characteristics of the subsoil. The buildings made of tufa and Lecce stone are those in which the largest concentrations of Radon were measured: it is therefore easily concluded that, in these municipalities of Lecce, the concomitant presence of many old buildings constructed with the abovementioned materials and the karst subsoil which affects the process of exhalation of Radon could, combined with smoking habits, be the cause of a significant number of lung neoplasms: at present, however, scientific feedback to this statement is required, especially bearing in mind that the spatial distribution of mortality is extremely different between the sexes, as we have well-established.

The presence of large industrial plants in the area of Galatina (Istat code: 75029, which borders the municipality of Cutrofiano, Istat code: 75026, located around the secondary cluster near the Maglie area), raises the question about the role of occupational exposure for male workers resident in central Salento: it would be interesting to know the employment and work place of males suffering from lung cancer to check, at least for areas classified as high risk, the possibility of an occupational exposure: furthermore, we must not forget the role that these facilities could have as point sources of pollutants and carcinogens diffused throughout the territory. These studies may become possible in the near future thanks to the consolidation of data from the Jonico-Salentino Cancer Register.

Despite the impressive figures concerning the environmental situation in the province of Lecce, one cannot conclude with certainty that the problem of environmental exposure caused by toxic substances released into the environment is directly responsible for the high lung cancer mortality registered in the province of Lecce: once again the differences between the sexes make it difficult to reach a similar conclusion. In addition, as already pointed out, deaths from lung cancer among males are above the Apulian average from as early as 1981, when the issue of emergency waste was probably much less relevant (if not entirely absent).

With regard to the conclusions of the local press that, as we have already mentioned in the introduction, has often attributed the causes of risk excess to air pollution produced by the factories of Taranto, it is noteworthy to observe that in support of these hypotheses it has often been cited a recent study on wind directions of Salento, which blow predominantly from the north-west, and therefore "would spread" atmospheric pollutants to west of the province of Lecce from the province of Taranto [71]. However, our conclusions disagree: the municipalities of Lecce province within the "Jonico-Salentina" belt (i.e. bordering the province of Taranto) are those with the lowest overall mortality rates (at least for males). The role of facilities for energy production installed in the province of Brindisi is unclear for similar reasons.

For females, given the situation observed in the city of Lecce, the enormous increase in mortality since the 90s is attributed to their clearly greater propensity to smoking: as the tobacco habit is a predominantly cultural phenomenon of nature, it can be assumed that many women, living in a city traditionally modern and cultured as Lecce, have gradually opted to smoke cigarettes as a symbol of an emancipated lifestyle. Even these findings, though plausible, are unfortunately weak because they were not derived from statistical models of incidence and/or mortality that take into account the latency period of lung cancer, and more precise data on cigarette consumption broken by municipality and sex: new studies will be needed to verify these new hypotheses.

For males, the presence of high levels of deprivation throughout the eastern and southern Salento is likely to play an important role: the most obvious explanation for the observed differentials is that those with lower socio-economic status smoke more [72], and that gender differences may be explained on the basis of the fact that in less developed areas women have less habit to tobacco smoking and alcohol drinking (and to other lifestyles harmful for health), which are seen as purely masculine behaviour. Of course, potential for uncontrolled confounding may be substantial, and the inquiry about the role of deprivation should be further developed and supported by data on individual lifestyles.

### Study limitations

The use of mortality rather incidence data, which could be a source of bias, was necessary by the fact that a methodical collection of cancer incidence data in the province of Lecce exists only from 1999 (thanks to the "Ionico-Salentino" Cancer Registry: on these issues, see the discussion reported in [73]). However, we have a substantial degree of confidence that these distortions can be regarded as negligible, given that roughly two-thirds of patients are diagnosed at an advanced stage of the disease, when available treatment approaches are rarely effective: as a consequence of the fact, lung cancer, especially non-small cell lung cancer, still appears as one of the more incurable neoplastic diseases, and that presents among the lowest five-year survival rates (see for example [74] showing the relative five-year survival rate – namely the relationship between the proportion of patients who survived in a cohort of individuals affected by lung cancer and the proportion of survivors expected in a comparable series of individuals not affected by the disease – equal to 11% for 2006). So, the quantitative dimensions of incidence and mortality cannot be considered too dissimilar: similar conclusion may be based on the recent data reported in [75].

The use of an areal approach invites the criticism of ecological fallacy [76], that is all individuals living in an area share the same characteristics, which is clearly not the case. In addition, the aggregate nature of the study made impossible to control for potential and very important confounders, such as smoking habits (for which neither aggregate or individual data were available) or occupational exposure among others. This implies that, notwithstanding the use of advanced disease mapping methods is essential to generate new etiological hypotheses, we have no possibility of ascertaining the real causes of risk excess clusters present in the data, and we can only make conjectures. To obtain information about the contribution of individuals to disease occurrence, and include within-area confounders, it is desirable to use both data collected on areas and on individuals [77]: this approach will be pursued in future papers as soon as individual-level data will become available.

## Conclusion

The data analyzed in this paper reveal that in Salento there are, unfortunately, all the adverse conditions for a high mortality for lung cancer, although the real reasons for this risk excess remain, until now, not fully understood: this work is a preliminary analysis that has served to estimate the spatial pattern of risk and to generate new hypotheses for study: research into the role of material deprivation and individual lifestyle differences between genders should be further developed: other findings, highlighted by the press, seem unrealistic in the light of data. The importance of geographical epidemiology as a tool for analyzing the dynamics and determinants of health and disease in the community was stressed.

## Competing interests

The authors declare that they have no competing interests.

## Authors' contributions

MB conceived the study, wrote sections 'Materials and methods' and 'Results', and revised the draft manuscript. AF wrote sections 'Abstract', 'Background', 'Discussion' and 'Conclusions'. Both authors read and approved the final manuscript

## Supplementary Material

Additional file 1 **ISTAT**. Amended list of municipalities in the province of Lecce, with its code extracted from the list of Italian municipalities published by ISTAT and updated on 1 January 2007. The municipal area of Racale has been combined with that of Taviano for the reasons discussed in the text. Click here for file
